# BMI Trajectories During the First 2 Years, and Their Associations With Infant Overweight/Obesity: A Registered Based Cohort Study in Taizhou, China

**DOI:** 10.3389/fped.2021.665655

**Published:** 2021-05-12

**Authors:** Tian Zhang, Ying Song, Haoyue Teng, Yue Zhang, Jianan Lu, Linghua Tao, Yanjie Jin, Jieyun Yin, Danhong Zhou

**Affiliations:** ^1^Department of Epidemiology and Biostatics, Jiangsu Key Laboratory of Preventive and Translational Medicine for Geriatric Diseases, School of Public Health, Medical College of Soochow University, Suzhou, China; ^2^Department of Gynaecology and Obstetrics, Taizhou Woman and Children's Hospital, Taizhou, China; ^3^Beijing Longtengxinyang Information Technology Company, Beijing, China

**Keywords:** BMI trajectory, infant, overweight, obesity, latent class growth mixture model

## Abstract

**Objective:** The purpose of this study was to identify trajectories of body mass index (BMI) in toddlers from birth to 2 years old and examine their association with infantile overweight/obesity.

**Methods:** Data were collected from 19,054 children born in any hospital or community healthcare center in Taizhou, China from 2018 to 2019 with at least three BMI measurements after birth. The Latent Class Growth Mixture Model was used to identify distinct BMI trajectories during the first 2 years of infants. Multiple logistic regression models were conducted to explore the associated factors of different BMI trajectories, and log-binomial regression was performed to assess the association between the trajectories and overweight/obesity.

**Results:** Three heterogeneous BMI trajectories were identified and labeled as “lower” (36.21%, *n* = 6,899), “middle” (53.15%, *n* = 10,128) and “upper” (10.64%, *n* = 2,027), respectively. Several characteristics of infants and their corresponding mothers were found to be correlated with infant BMI trajectories, including infant sex, mode of delivery and weight at birth, as well as maternal parity, early pregnancy BMI and status of gestational diabetes mellitus. Furthermore, compared with those in the lower trajectory, infants in the middle [prevalence ratio (PR) = 2.63, 95% confidence interval (95%CI) = 2.17–2.63] or upper (PR = 2.98, 95%CI = 1.51–2.98) trajectory groups were prone to be overweight/obesity at their final observation.

**Conclusion:** Heterogeneous BMI trajectories were observed in our study. Characteristics of both infants and their corresponding mothers could be potential determinants of infant growth. Moreover, infants in the middle and upper trajectory groups were more likely to suffer overweight/obesity.

## Introduction

Overweight and Obesity, defined by the World Health Organization (WHO) as an abnormal or excessive fat accumulation, present an alarming risk to health (1). Childhood overweight and obesity have become a major public health concern (2). In China, a national survey reported that the prevalence of overweight and obesity in children aged 7–17 years had increased rapidly from 3.8% and 0.6% in 1995 to 14.3% and 4.1% in 2014, respectively (3). Excessive weight gain in early life is likely to lead to lifelong overweight and obesity (4). Meanwhile, childhood overweight/obesity is associated with greater risk and earlier onset of chronic disorders, such as heart disease, asthma, type 2 diabetes, and psychosocial disorders (5–7). Thus, the early identification and intervention of overweight/obesity in infants is critical to their long-term health conditions.

Body mass index (BMI) is widely accepted to assess children's weight status. As we known, BMI fluctuates in early life (8). In general, BMI rapidly increases during the 1st year after birth, then decreases until reaching a nadir around 6 years of age (also called the adiposity rebound); thereafter, BMI increases again throughout childhood and finally reaches a plateau late in life (8). At the population level, BMI and changes in BMI during childhood are highly heterogeneous (9, 10). Recent evidence has suggested that there are varied BMI changing patterns among children, which may bring different health outcomes (10, 11). Hence, BMI at a single occasion may be insufficient to explain the effects of obesity on disease (12). To better describe the dynamic change of BMI in early life and its influence on health conditions, the BMI trajectory has been introduced. Latent class growth mixture model (LCGMM) is particularly useful for capturing the changing pattern within a certain period of time, as it allows simultaneous estimation of multiple trajectories (10, 13, 14).

In view of this, the identification of different BMI trajectories and their associated factors in children may be helpful for their health (15, 16). According to Barker's fetal origins of disease hypothesis (17), we hypothesized that prenatal, neonatal and infantile factors may influence the BMI trajectory during infancy. Hence, comprehensively assessment of possible influential factors and establishment of prompt management programs guided by trained doctors and other health care providers through pregnancy and infancy, is of great value for preventing and controlling infantile obesity. However, most studies shed light in this field were carried in developed countries (18–20), and evidence in developing countries is still scarce.

Therefore, our study which based on a Chinese infant cohort from Taizhou City, Zhejiang Province, sought to reach the following research objectives: (1) to identify the latent BMI growth trajectories from birth to 2 years by conducting LCGMM; (2) to explore the associated factors of the identified BMI trajectories; (3) to examine the associations between different trajectories of BMI and overweight/obesity in infancy.

## Methods

### Study Population

**Figure 1:**
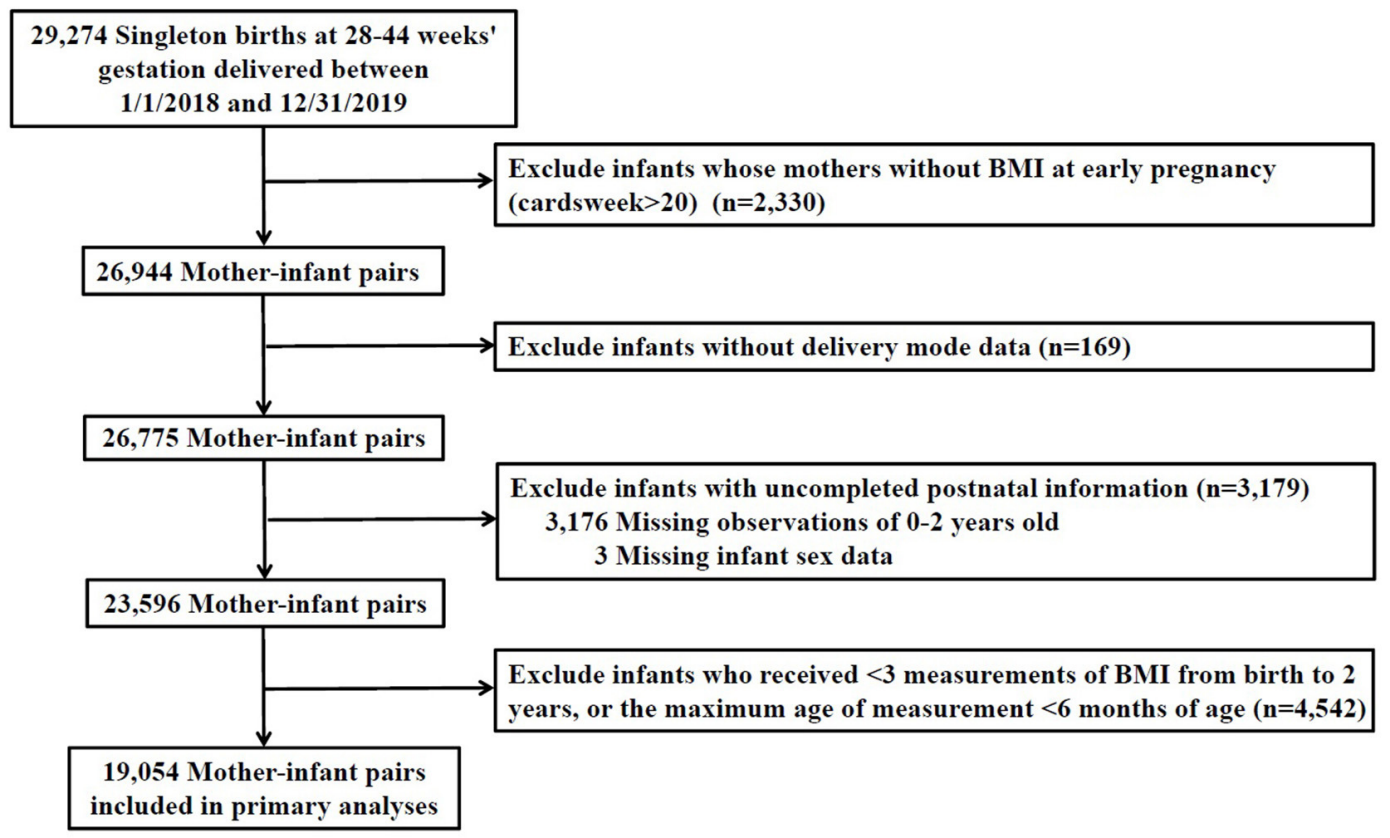
Flow chart for selection process of the study.

A total of 29,274 singleton children who born in any hospital or community healthcare center in Taizhou city from 01/01/2018-31/12/2019 were recruited in our study. Information for children and their corresponding mothers were collected through questionnaires or medical records. First, exclusion was made for participants with uncompleted information of prenatal characteristics [maternal BMI at early stage of pregnancy (*n* = 2,330)]. Second, we excluded infants with missing intrapartum [mode of delivery (*n* = 169)] or postnatal data [missing for follow-up during 0–2 years old (*n* = 3,176) and without gender information (*n* = 3)], resulting in a total of 23,596 participants. Third, we excluded 4,542 infants who received <3 measurements of BMI from birth to 2 years and whose age of last measurement was <6 months. Ultimately, a total of 19,054 mother-child pairs were included. The flowchart of the exclusion and inclusion process of our study population is presented in Figure 1.

### Infant and Maternal Anthropometric Data

Weight and length were measured by trained nurses using standardized techniques with subjects in light clothing and without shoes. Infant weight was measured using a digital scale to the nearest 0.1 kg and length was measured from the top of head to soles of feet using an infant calibrated measuring mat to the nearest 0.1 cm. Infantile BMI at the last observation was adopted to determine the status of overweight or obese. Based on WHO growth standards, an infant whose BMI > 95th or between 85 and 95th percentiles for each sex and month were defined to be obese and overweight, respectively (21). Maternal weight at early stage of pregnancy (in the 1st trimester) was measured by doctors at their first prenatal visit to the nearest 0.1 kg in light clothing by using the calibrated beam scale, and maternal height was measured to the nearest 0.1 cm using a portable stadiometer. An average of three times measurements for infant and maternal weight/height was recorded.

### Details of Maternal/Infantile Characteristics

Information for mother-child pairs was firstly collected through questionnaire, including (1) maternal demographics, such as age, education level, parity (primiparous/multiparous) and abortion times; (2) prenatal characteristics, including early pregnancy BMI, smoking and drinking status during pregnancy; (3) infantile characteristics, such as infant feeding styles, daily intake of vitamin D, and outdoor activity duration. Education was divided into four levels: primary school or below, junior school, high school, university or above. Maternal age at delivery was divided into four categories (<18, 18–24, 25–34, and ≥35 years). Maternal feeding styles were classified into two groups, as exclusive breastfeeding or not. Daily intake of vitamin D was divided into three dosages (0–400, 400–600, >600 IU/d). Outdoor activity duration was divided into three levels (0–1, 1–2, >2 h/day).

Other maternal characteristics were then retrieved from medical records. Maternal BMI at early pregnancy was classified following Chinese BMI classification standard as lean (BMI <18.5 kg/m^2^), normal (18.5 ≤ BMI ≤ 23.9 kg/m^2^), overweight (24 ≤ BMI ≤ 27.9 kg/m^2^) and obese (BMI ≥ 28 kg/m^2^) (22). Gestational complications mainly included gestational diabetes, gestational hypertension, anemia, and thyroid disease.

Infant birth weight, gender, gestational age at delivery, delivery mode and Apgar scores were also extracted from medical records. The gestational age is measured by the last menstrual period or ultrasound (23). Mode of delivery was divided into two categories (vaginal delivery and cesarean). Small for gestational age infant (SGA) was defined as the infant whose birth weight lower than the 10th centile for a specific completed gestational age by gender. The Apgar score of 8–10 is considered normal (24).

### Identification of BMI Trajectories

In our study, the change of patterns of BMI trajectories was fitted by LCGMM by using Proc Traj in SAS 9.4. A censored normal model was considered appropriate due to the continuity of BMI. For each model specifying a given number of trajectories, we tested linear, quadratic, and cubic equations. The smaller the Bayesian Information Criterion (BIC) value, the better the model fits the data. For each model involving latent classes, posterior class-membership probabilities (PP) were also used to obtain a posterior classification of the participants in each latent class to evaluate goodness-of-fit and to characterize the discrimination of latent clusters. We further retrieved the percentage of subjects classified in each class with a PP above a threshold of 0.7, indicating the proportion of subjects unambiguously classified in each latent class (13). These procedures indicated that a model with three trajectories (with cubic curve assumption) was superior to models with fewer trajectories because of much smaller BIC values. Although models with 4 or 5 trajectories had smaller BIC values than the three trajectories, the former models yielded trajectories with <5% of participants or the percentage of mean PP >0.7 <65% (13). Models with more than five groups were not explored for their small PP or big BIC values. The parameter estimates of the models with 1 to 5 trajectories were given in Supplementary Table 1.1. Therefore, further analyses were based on the three trajectories with cubic curve assumption.

### Statistical Analysis

Maternal and infant characteristics were compared among different latent BMI trajectory groups by using chi-square test for proportions and analysis of variance (ANOVA) test for means, respectively. Association between prenatal and early life factors with middle and upper BMI trajectories in comparison to the lower trajectory group were conducted by logistic regression models. Afterwards, four log-binomial regression models were used to explore the association between childhood obesity/overweight and BMI trajectories. Model 1 was a crude model. Model 2 was adjusted for sex, infant's age at the last observation, and maternal education level. Model 3 additionally adjusted for maternal factors, including parity, early pregnancy BMI, gestational diabetes mellitus (GDM), and gestational hypertension. Based on Model 3, model 4 further included infants' characteristics, such as mode of delivery, outdoor activity duration, maternal feeding styles, SGA, infant age at the final observation, and intake of vitamin D. At last, subgroups analyses by sex were conducted to see whether there are any gender differences. All statistical tests were performed using SAS software (version 9.4, SAS Institute, Cary, NC, USA), and differences were considered statistically significant with two-sided *P* ≤ 0.05.

## Results

### BMI Trajectories

**Figure 2:**
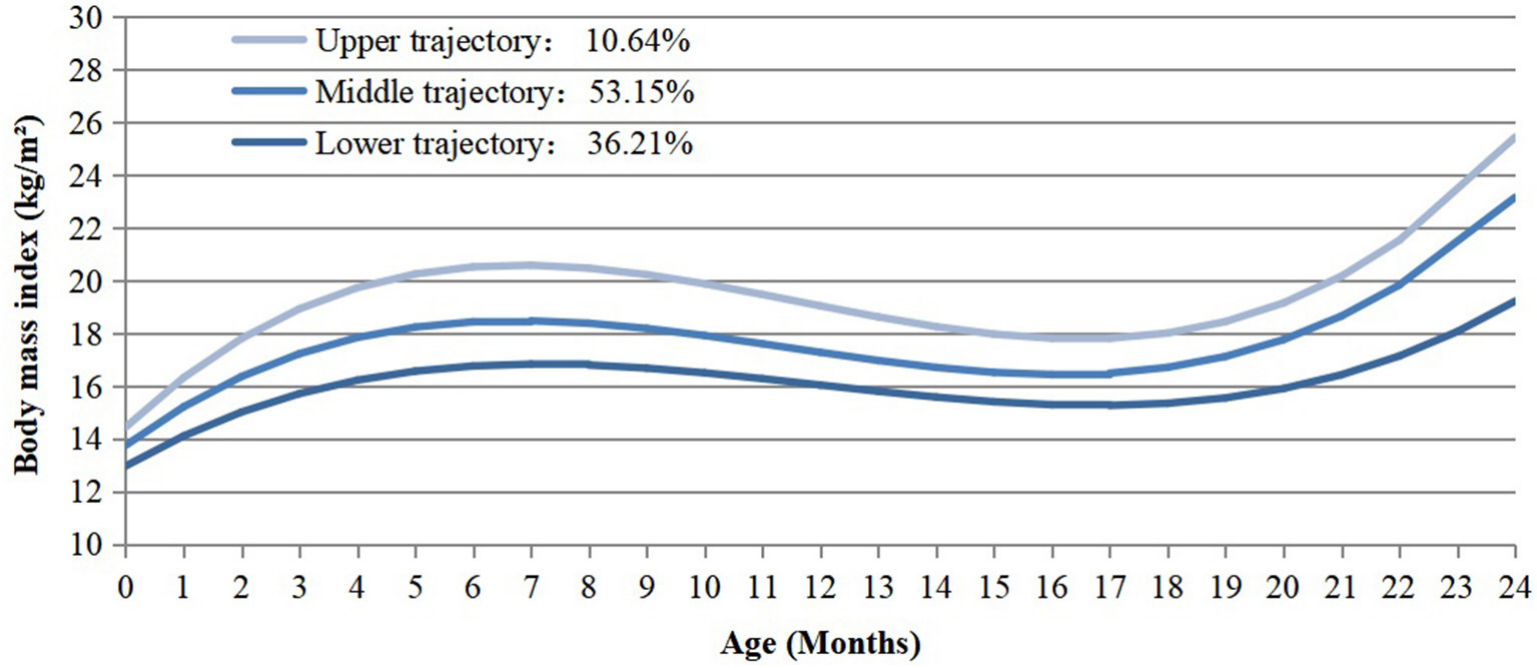
BMI trajectories from birth to 2 years old identified from the LCGMM.

A total of 19,054 singleton children delivered at 28–44 weeks of gestation were enrolled in our study. The infants had a total of 101,251 BMI measurements since birth, the median measurement times of each infant was 5 (interquartile range, 4–6). Three distinct BMI trajectory groups were identified (Figure 2). They were labeled as “lower” (*n* = 6,899, 36.21%), “middle” (*n* = 10,128, 53.15%) and “upper” (*n* = 2,027, 10.64%), respectively. The lower trajectory started with relatively low BMI (12.97 kg/m^2^), then followed with a steady acceleration until the age of seven months (16.83 kg/m^2^). After peaking at 7 months, it declined gradually and reached the lowest point at 17 months, followed by an increase subsequently. The middle trajectory started with a mid-level BMI (13.75 kg/m^2^), while the upper one showed a higher level of BMI (14.44 kg/m^2^) at beginning. Both of them reached the climax at 7 months (“middle” = 18.47 kg/m^2^, “upper” = 20.58 kg/m^2^) and the lowest point at 17 months after birth, which were parallel to the process of the lower one. The slope and intercept of the specific trajectories were present in Supplementary Table 1.2.

### Baseline Characteristics

Characteristics of infants and their mothers were illustrated in Table 1. In the included population, maternal age ranged between 14 and 49 years old [mean = 30.61, standard deviation (SD) = 5.22]. Mothers who received education of high school or above accounted for 67.15%. A slightly larger number of mothers gave birth to a male infant (52.34%) or delivered by cesarean section (51.02%). Few mothers drank (0.18%) or smoked (0.10%) during pregnancy. Besides, the mean birth weight of the infants was 3320.30 (SD = 393.87) grams and the mean gestational age at delivery was 38.99 (SD = 1.24) weeks. A total of 2,994 (15.71%) infants were identified as overweight and 1,821 (9.56%) became obese at their final observation. Meanwhile, infants born from mothers with multiparity, higher BMI at early pregnancy, or GDM were more likely to be in the upper group. Also, infantile characteristics such as gender, gestational age, birth weight, and outdoor time differed among three trajectory groups.

**Table 1:** Characteristics of mother-child pairs by BMI trajectories.

**Variables**	**Total population**	**Lower trajectory**	**Middle trajectory**	**Upper trajectory**	***P*-value**
	***N* = 19,054**	***n* = 6,899**	***n* = 10,128**	***n* = 2,027**	
**Maternal characteristics**					
Maternal demographics					
Age (years) (mean ± SD)	30.61 ± 5.22	30.56 ± 5.31	30.66 ± 5.20	30.56 ± 4.99	0.415
Education, *n* (%)					0.123
Primary school or below	1,201 (6.30%)	474 (6.87%)	617 (6.09%)	110 (5.43%)	
Junior school	5,059 (26.55%)	1,826 (26.47%)	2,664 (26.30%)	569 (28.07%)	
High school	4,892 (25.68%)	1,739 (25.21%)	2,639 (26.06%)	514 (25.36%)	
University or above	7,902 (41.47%)	2,860 (41.45%)	4,208 (41.55%)	834 (41.14%)	
Parity, *n* (%)					** <0.001**
Primiparous	6,736 (35.35%)	2,614 (37.89%)	3,468 (34.24%)	654 (32.26%)	
Multiparous	12,318 (64.65%)	4,285 (62.11%)	6,660 (65.76%)	1,373 (67.74%)	
Abortion times, *n* (%)					
Induced					0.397
0	13,140 (68.96%)	4,783 (69.33%)	6,957 (68.69%)	1,400 (69.07%)	
1	3,403 (17.86%)	1,219 (17.67%)	1,803 (17.80%)	381 (18.80%)	
2+	2,511 (13.18%)	897 (13.00%)	1,368 (13.51%)	246 (12.13%)	
Spontaneous					0.762
0	17,854 (93.70%)	6,475 (93.85%)	9,480 (93.60%)	1,899 (93.68%)	
1	993 (5.21%)	353 (5.12%)	530 (5.23%)	110 (5.43%)	
2+	207 (1.09%)	71 (1.03%)	118 (1.17%)	18 (0.89%)	
Prenatal characteristics					
BMI at early stage of pregnancy, *n* (%)					** <0.001**
BMI < 18.5 kg/m^2^	3,412 (17.91%)	1,629 (23.61%)	1,558 (15.38%)	225 (11.10%)	
BMI:18.5–23.9 kg/m^2^	11,042 (57.95%)	3,991 (57.85%)	5,931 (58.56%)	1,120 (55.26%)	
BMI:24.0–27.9 kg/m^2^	3,631 (19.06%)	1,039 (15.06%)	2,072 (20.46%)	520 (25.65%)	
BMI ≥ 28.0 kg/m^2^	969 (5.08%)	240 (3.48%)	567 (5.60%)	162 (7.99%)	
Smoking during pregnancy, *n* (%)	20 (0.10%)	6 (0.09%)	12 (0.12%)	2 (0.10%)	0.820
Drinking during pregnancy, *n* (%)	34 (0.18%)	7 (0.10%)	22 (0.22%)	5 (0.25%)	0.159
Gestational week (mean ± SD)	38.99 ± 1.24	38.93 ± 1.31	39.02 ± 1.20	39.04 ± 1.19	** <0.001**
Gestational complications, *n* (%)					
GDM	2,795 (14.67%)	995 (14.42%)	1,464 (14.45%)	336 (16.58%)	**0.037**
Gestational hypertension	300 (1.57%)	121 (1.75%)	149 (1.47%)	30 (1.48%)	0.325
Anemia	3,051 (16.01%)	1,107 (16.05%)	1,647 (16.26%)	297 (14.65%)	0.196
Thyroid disease	2,618 (13.74%)	933 (13.52%)	1,378 (13.61%)	307 (15.15%)	0.149
**Infantile characteristics**					
Neonatal characteristics					
Sex, *n* (%)					** <0.001**
Female	9,081 (47.66%)	4,481 (64.95%)	4,103 (40.51%)	497 (24.52%)	
Male	9,973 (52.34%)	2,418 (35.05%)	6,025 (59.49%)	1,530 (75.48%)	
SGA, *n* (%)	1,658 (8.70%)	1,161 (16.83%)	469 (4.63%)	28 (1.38%)	** <0.001**
Mode of delivery, *n* (%)					** <0.001**
Cesarean	9,722 (51.02%)	3,260 (47.25%)	5,248 (51.82%)	1,214 (59.89%)	
Vaginal delivery	9,332 (48.98%)	3,639 (52.75%)	4,880 (48.18%)	813 (40.11%)	
Apgar at 1 min ≥ 8, *n* (%)	18,880 (99.09%)	6,827 (98.96%)	10,041 (99.14%)	2,012 (99.26%)	0.317
Apgar at 5 mins ≥ 8, *n* (%)	19,010 (99.77%)	6,882 (99.75%)	10,109 (99.81%)	2,019 (99.61%)	0.196
Birth weight (g) (mean ± SD)	3320.30 ± 393.87	3132.25 ± 376.47	3392.20 ± 397.82	3601.09 ± 430.28	** <0.001**
Postnatal characteristics					
Exclusive breastfeeding, *n* (%)	12,214 (64.10%)	4,372 (63.37%)	6,498 (64.16%)	1,344 (66.30%)	0.053
Daily intake of vitamin D (IU/d), *n* (%)					0.254
0 ≤ vitamin D ≤ 400	16,423 (86.19%)	5,942 (86.13%)	8,736 (86.26%)	1,745 (86.09%)	
400 < vitamin D ≤ 600	2,193 (11.51%)	777 (11.26%)	1,175 (11.60%)	241 (11.89%)	
600 < vitamin D	438 (2.30%)	180 (2.61%)	217 (2.14%)	41 (2.02%)	
Outdoor activity duration (hours/d), *n* (%)					**0.002**
0–1	424 (2.23%)	138 (2.00%)	237 (2.34%)	49 (2.42%)	
1–2	5,391 (28.29%)	1,890 (27.40%)	2,861 (28.25%)	640 (31.57%)	
≥2	13,239 (69.48%)	4,871 (70.60%)	7,030 (69.41%)	1,338 (66.01%)	
Overweight, *n* (%)	2,994 (15.71%)	95 (1.38%)	2,278 (22.49%)	621 (30.64%)	** <0.001**
Obesity, *n* (%)	1,821 (9.56%)	9 (0.13%)	647 (6.39%)	1,165 (57.47%)	** <0.001**

*Note: Boldface indicates statistical significance (p < 0.05)*.

### Predictors of BMI Trajectories

Table 2 presents the associations between prenatal as well as infantile factors and their growth trajectories. Birth to a multiparous mother [odds ratio (OR) = 1.28, 95% confidence interval (95%CI) = 1.15–1.42] was a risk factor for being in the upper trajectory group. Maternal obesity (BMI ≥ 28 kg/m^2^) at early stage of pregnancy (OR = 2.41, 95%CI = 1.95–2.97) and complicated with GDM (OR = 1.18, 95%CI = 1.03–1.35) were also associated with increased risk of belonging to the upper trajectory. Besides, the highest BMI groups tended to include infants who had exclusive breastfeeding (OR = 1.14, 95%CI = 1.03–1.26) or male gender (OR = 5.71, 95%CI = 5.10–6.39). However, vaginally delivered (OR = 0.60, 95%CI = 0.54–0.66) and SGA (OR = 0.07, 95%CI = 0.05–0.10) infants were less likely to be grouped into the upper trajectory.

**Table 2:** Association between prenatal and early life factors with middle and upper BMI trajectories in comparison to the lower trajectory group.

**Variables**	**Middle trajectory**	**Upper trajectory**
	**OR (95%CI)**	**OR (95%CI)**
**Maternal characteristics**		
Maternal demographics		
Age (years)		
<18	0.62 (0.31–1.26)	0.20 (0.03–1.50)
18–24	**0.89 (0.81**–**0.98)**	**0.85 (0.73**–**0.99)**
25–34	1.00 (reference)	1.00 (reference)
≥35	0.95 (0.88–1.02)	**0.83 (0.73**–**0.93)**
Education		
Primary school or below	1.00 (reference)	1.00 (reference)
Junior school	1.12 (0.98–1.28)	**1.34 (1.07**–**1.69)**
High school	**1.17 (1.02**–**1.33)**	**1.27 (1.01**–**1.60)**
University or above	1.13 (0.99–1.29)	**1.26 (1.01**–**1.57)**
Prenatal characteristics		
BMI at early stage of pregnancy		
BMI < 18.5 kg/m^2^	**0.64 (0.59**–**0.70)**	**0.49 (0.42**–**0.57)**
BMI: 18.5–23.9 kg/m^2^	1.00 (reference)	1.00 (reference)
BMI: 24.0–27.9 kg/m^2^	**1.34 (1.23**–**1.46)**	**1.78 (1.58**–**2.02)**
BMI ≥ 28.0 kg/m^2^	**1.59 (1.36**–**1.86)**	**2.41 (1.95**–**2.97)**
Parity		
Primiparous	1.00 (reference)	1.00 (reference)
Multiparous	**1.17 (1.10**–**1.25)**	**1.28 (1.15**–**1.42)**
Smoking		
No	1.00 (reference)	1.00 (reference)
Yes	1.36 (0.51–3.63)	1.14 (0.23–5.63)
Drinking		
No	1.00 (reference)	1.00 (reference)
Yes	2.14 (0.91–5.00)	2.43 (0.77–7.65)
Gestational complications		
GDM		
No	1.00 (reference)	1.00 (reference)
Yes	1.00 (0.92–1.09)	**1.18 (1.03**–**1.35)**
Gestational hypertension		
No	1.00 (reference)	1.00 (reference)
Yes	0.84 (0.66–1.07)	0.84 (0.56–1.26)
Anemia		
No	1.00 (reference)	1.00 (reference)
Yes	1.02 (0.94–1.10)	0.90 (0.78–1.03)
Thyroid disease		
No	1.00 (reference)	1.00 (reference)
Yes	1.01 (0.92–1.10)	1.14 (0.99–1.31)
**Infant characteristics**		
Neonatal characteristics		
Sex		
Female	1.00 (reference)	1.00 (reference)
Male	**2.72 (2.55**–**2.90)**	**5.71 (5.10**–**6.39)**
SGA		
No	1.00 (reference)	1.00 (reference)
Yes	**0.24 (0.22**–**0.27)**	**0.07 (0.05**–**0.10)**
Mode of delivery		
Cesarean	1.00 (reference)	1.00 (reference)
Vaginal delivery	**0.83 (0.78**–**0.89)**	**0.60 (0.54**–**0.66)**
Apgar at 1 min		
≥8	1.00 (reference)	1.00 (reference)
<8	0.82 (0.60–1.12)	0.71 (0.40–1.24)
Apgar at 5 mins		
≥8	1.00 (reference)	1.00 (reference)
<8	0.76 (0.40–1.47)	1.60 (0.69–3.72)
Postnatal characteristics		
Exclusive breastfeeding		
No	1.00 (reference)	1.00 (reference)
Yes	1.04 (0.97–1.10)	**1.14 (1.03**–**1.26)**
Vitamin D (IU/d)		
0 ≤ Vitamin D ≤ 400	1.00 (reference)	1.00 (reference)
400 < Vitamin D ≤ 600	1.03 (0.93–1.13)	1.06 (0.91–1.23)
Vitamin D > 600	0.82 (0.67–1.00)	0.78 (0.55–1.09)
Outdoor activity duration (hours/day)		
0–1	1.00 (reference)	1.00 (reference)
1–2	0.88 (0.71–1.10)	0.95 (0.68–1.34)
≥2	0.84 (0.68–1.04)	0.77 (0.56–1.08)

*Note: Boldface indicates statistical significance (p < 0.05)*.

### Association Between BMI Trajectories and Infant Overweight/Obesity

As shown in Table 3, 1.51, 28.88, and 88.11% of participants were overweight/obesity in the lower, the middle and the upper trajectory groups, respectively. In comparison with children in the lower trajectory, children in the middle trajectory group [prevalence ratio (PR) = 19.16, 95%CI = 15.89–23.39] and the upper trajectory group [PR = 58.45, 95% CI = 48.56–71.23] were at a higher risk to be overweight/obesity at the age of their final observation in the crude model. After adjusting for co-variables in Models 2–4, the significance remained.

**Table 3:** Results from log-binomial regression of the associations between childhood obesity/overweight and BMI trajectories.

	**Lower trajectory**	**Middle trajectory**	**Upper trajectory**
	***n* = 6,899**	***n* = 10,128**	***n* = 2,027**
	**PR (95%CI)**	**PR (95%CI)**	**PR (95%CI)**
Overweight/Obesity (*n*, %)	104 (1.51%)	2,925 (28.88%)	1,786 (88.11%)
Model 1^*^	1.00 (reference)	19.16 (15.89–23.39)	58.45 (48.56–71.23)
Model 2^*^	1.00 (reference)	19.89 (16.49–24.28)	60.54 (50.31–73.76)
Model 3^*^	1.00 (reference)	5.33 (5.29–13.24)	10.74 (10.73–10.75)
Model 4^*^	1.00 (reference)	2.63 (2.17–2.63)	2.98 (1.51–2.98)

^*^Model 1 was unadjusted.

^*^Model 2 was adjusted for age, gender, education.

^*^Model 3 was additionally controlled for maternal factors, including parity, early pregnancy BMI, GDM, gestational hypertension, based on model 2.

^*^Model 4 was additionally controlled for characteristics of infants, including mode of delivery, outdoor activity duration, maternal feeding styles, SGA, infant age of their last measurement and daily intake of vitamin D, based on model 3.

### Subgroup Analysis by Gender

BMI trajectories were also explored in separated gender groups. As shown in Supplementary Figure 1, BMI trajectory curves were generally similar between girls and boys. Supplementary Tables 2.1, 2.2 present the characteristics of mother-infant pairs among different BMI trajectories in boys and girls, respectively. Several maternal (i.e., parity, BMI at early pregnancy, and gestational age at delivery) and infantile characteristics (such as SGA, mode of delivery and infantile birth weight) were possible determining factors of the identified BMI trajectories in both genders.

With the lower group as a reference, Supplementary Tables 3.1, 3.2 present the associations between prenatal/early life factors with middle and upper BMI trajectories in boys and girls, respectively. For both boys and girls, born from a multiparous mother, higher maternal BMI at early pregnancy were risk factors for belonging to the upper trajectory group. As shown in Supplementary Tables 4.1, 4.2, both male and female infants in the middle trajectory group and upper trajectory group were more likely to suffer overweight/obesity, which were similar to the results for the whole population.

## Discussion

Our study found that there are three discrete BMI trajectories of infant growth from birth to 2 years old, and infants in the middle and upper trajectories are more likely to be overweight and obese compared with those in the lower group. Besides, we identified several potential determining factors for BMI trajectories, including maternal demographics (age at delivery, education level, parity), prenatal diseases (GDM), prenatal (BMI at early stage of pregnancy), neonatal (sex, birth weight, mode of delivery) and postnatal characteristics (maternal feeding styles).

Our study provided an insight of associations between maternal prenatal factors and offspring BMI trajectories. Our finding that multiparity was associated with an increased risk of a child with higher BMI, which was consistent with previous findings (25, 26). Additionally, our results also supported the view that mothers being obese at the early stage of pregnancy may increase the risk of obesity of their offspring (27). A prior study reported that maternal obesity in early pregnancy is the most important factor differentiating BMI trajectories (26). It was suggested that maternal weight represented the nutritional environment of the fetus. To adapt to the environment of over nutrition, the fetus formed a certain fat tissue memory system, which led to the phenomenon that an infant would be over-weighted since he was born and may suffer from the permanent high weight throughout his whole life (4). It was also found that infants whose mothers had received higher-level education tended to be classified into the middle or upper BMI trajectory. It could be explained that a higher level of maternal education is correlated with a better quality of diet and a more adequate intake of various nutrients for children (28). We also examined the influence of intrapartum factors on offspring BMI trajectories. Similar to previous studies (29, 30), cesarean section was linked with higher odds of children with higher BMI. Compared with infants born vaginally, those born by cesarean section have less bifidobacteria, and less diverse bacteria species in microbiota colonization, a pattern that has been linked with increased capacity for energy harvest and risk of overweight and obesity in later life (31, 32). Besides, cesarean section may have a great impact on infant appetite regulation and energy metabolism than vaginal delivery, which may contribute to the significant increase in body mass (33). Additionally, our results were consistent with previous studies showing that maternal status of GDM as well as infant male gender play an important role in the progression of childhood obesity (34, 35). Maternal gestational hyperglycemia may predispose offspring to increased adiposity, impaired glucose tolerance, hyperinsulinemia, and insulin resistance, which leads to offspring obesity (36).

Our study also identified exclusive breastfeeding as a risk factor of being in the upper BMI trajectory. However, some prior studies suggested that exclusive breastfeeding was a protective factor for infant obesity, since the lower protein content of breast milk (in comparison to formula milk) may reduce adipocyte development (37, 38). Nevertheless, it is still controversial whether exclusive breastfeeding could influence the possibility of rising infant weight gain patterns (39), and the underlying mechanisms remain unclear.

Strengths of our study lie in the relatively large sample size and the continuity of infant growth period up to 2 years old. Studies focusing on BMI trajectories were mainly children from western countries (18–20). Regional differences may exist in BMI trajectories which vary in terms of dimensions, elevation, living habits, dietary habits, and socio-economic status. The participants in our study come from Taizhou, a south-eastern coastal city located in Zhejiang Province, China. The geographic location of the city is 120°34' E and 28°50' N. It has more than 5.97 million permanent residents, features a booming economy and high population density. Given the paucity of data on growth trajectories in Asian population, our study enriched the field and may help develop intervention strategies to control the rapidly increasing prevalence of childhood obesity.

There remained some limitations in our current study. Firstly, other potential confounding factors, such as paternal characteristics, genetics, environmental and dietary habits (40), were not assessed in the current study. Secondly, due to the nature of observational study, we cannot infer the causal relationship between those possible risk factors and changes in infant BMI. Thirdly, children included in our study are from Taizhou city in China, and thus may not be representative for other parts of China and other nationalities. Considering these limitations, further studies are needed.

## Conclusion

Three distinct latent BMI trajectories were identified for children from birth to 2 years old including the lower, middle and upper groups. Infants in the middle and upper trajectory groups are more likely to be obese late in their life. Our results also underline the importance of the timely intervention on children overweight/obesity targeted at potential risk factors during both prenatal and postnatal period.

## Data Availability Statement

The raw data supporting the conclusions of this article will be made available by the authors, without undue reservation.

## Ethics Statement

The studies involving human participants were reviewed and approved by ethic committee of Medical College of Soochow University. Written informed consent to participate in this study was provided by the participants' legal guardian/next of kin. Written informed consent was obtained from the individual(s), and minor(s)' legal guardian/next of kin, for the publication of any potentially identifiable images or data included in this article.

## Author Contributions

JY and DZ has contributed to the design and concept of the manuscript. TZ and YS were responsible for the analysis, interpretation of data and manuscript drafting. HT, YZ, JL, LT, and YJ were responsible for critical revision of the manuscript for intellectual content. All authors were involved in writing the paper and had final approval of the submitted and published versions.

## Conflict of Interest

The authors declare that the research was conducted in the absence of any commercial or financial relationships that could be construed as a potential conflict of interest.

## Abbreviations

WHO: World Health Organization
BMI: Body mass index
LCGMM: latent class growth mixture model
SGA: small for gestational age
BIC: Bayes information criterion
PP: Posterior probabilities
ANOVA: analysis of variance
GDM: gestational diabetes mellitus
SD: standard deviation
OR: odds ratio
95% CI: 95% confidence interval
PR: prevalence ratio.

## References

[1] WHO. Obesity. (2020). Available online at: https://www.who.int/health-topics/obesity#tab=tab_1 (accessed June 15, 2020).

[2] WHO. UNICEF/WHO/The World Bank Group Joint Child Malnutrition Estimates: Levels and Trends in Child Malnutrition: Key Findings of the 2020 Edition. (2020). Available from: https://www.who.int/publications/i/item/jme-2020-edition (accessed March 31, 2020).

[3] Dong Y Ma J Song Y Ma Y Dong B Zou Z. Secular trends in blood pressure and overweight and obesity in Chinese boys and girls aged 7 to 17 years from 1995 to 2014. Hypertension. (2018) 72:298–305. 10.1161/HYPERTENSIONAHA.118.11291 29866739 PMC6043402

[4] Weiss R Dziura J Burgert TS Tamborlane WV Taksali SE Yeckel CW. Obesity and the metabolic syndrome in children and adolescents. N Engl J Med. (2004) 350:2362–74. 10.1056/NEJMoa031049 15175438

[5] Bendor CD Bardugo A Pinhas-Hamiel O Afek A Twig G. Cardiovascular morbidity, diabetes and cancer risk among children and adolescents with severe obesity. Cardiovasc Diabetol. (2020) 19:79. 10.1186/s12933-020-01052-1 32534575 PMC7293793

[6] Skinner AC Perrin EM Moss LA Skelton JA. Cardiometabolic risks and severity of obesity in children and young adults. N Engl J Med. (2015) 373:1307–17. 10.1056/NEJMoa1502821 26422721

[7] Lang JE Bunnell HT Hossain MJ Wysocki T Lima JJ Finkel TH. Being overweight or obese and the development of asthma. Pediatrics. (2018) 142. 10.1542/peds.2018-2119 30478238

[8] Rolland-Cachera MF Deheeger M Maillot M Bellisle F. Early adiposity rebound: causes and consequences for obesity in children and adults. Int J Obes. (2006) 30(Suppl. 4):S11–7. 10.1038/sj.ijo.0803514 17133230

[9] Sun J Xi B Yang L Zhao M Juonala M Magnussen CG. Weight change from childhood to adulthood and cardiovascular risk factors and outcomes in adulthood: a systematic review of the literature. Obes Rev. (2021) 22:e13138. 10.1111/obr.13138 32875696

[10] Mattsson M Maher GM Boland F Fitzgerald AP Murray DM Biesma R. Group-based trajectory modelling for BMI trajectories in childhood: a systematic review. Obes Rev. (2019) 20:998–1015. 10.1111/obr.12842 30942535

[11] Rolland-Cachera MF Deheeger M Bellisle F Sempe M Guilloud-Bataille M Patois E. Adiposity rebound in children: a simple indicator for predicting obesity. Am J Clin Nutr. (1984) 39:129–35. 10.1093/ajcn/39.1.129 6691287

[12] Simmonds M Burch J Llewellyn A Griffiths C Yang H Owen C. The use of measures of obesity in childhood for predicting obesity and the development of obesity-related diseases in adulthood: a systematic review and meta-analysis. Health Technol Assess. (2015) 19:1–336. 10.3310/hta19430 26108433 PMC4781104

[13] Buscot MJ Thomson RJ Juonala M Sabin MA Burgner DP Lehtimaki T. Distinct child-to-adult body mass index trajectories are associated with different levels of adult cardiometabolic risk. Eur Heart J. (2018) 39:2263–70. 10.1093/eurheartj/ehy161 29635282

[14] Ziyab AH Karmaus W Kurukulaaratchy RJ Zhang H Arshad SH. Developmental trajectories of Body Mass Index from infancy to 18 years of age: prenatal determinants and health consequences. J Epidemiol Community Health. (2014) 68:934–41. 10.1136/jech-2014-203808 24895184 PMC4174013

[15] Barker DJ Osmond C. Infant mortality, childhood nutrition, and ischaemic heart disease in England and Wales. Lancet. (1986) 1:1077–81. 10.1016/S0140-6736(86)91340-1 2871345

[16] Weng SF Redsell SA Swift JA Yang M Glazebrook CP. Systematic review and meta-analyses of risk factors for childhood overweight identifiable during infancy. Arch Dis Child. (2012) 97:1019–26. 10.1136/archdischild-2012-302263 23109090 PMC3512440

[17] Barker DJ. The fetal origins of coronary heart disease. Eur Heart J. (1997) 18:883–4. 10.1093/oxfordjournals.eurheartj.a015368 9183571

[18] Nedelec R Miettunen J Mannikko M Jarvelin MR Sebert S. Maternal and infant prediction of the child BMI trajectories; studies across two generations of Northern Finland birth cohorts. Int J Obes. (2021) 45:404–14. 10.1038/s41366-020-00695-0 33041325

[19] Ni Y Beckmann J Hurst JR Morris JK Marlow N. Size at birth, growth trajectory in early life, and cardiovascular and metabolic risks in early adulthood: EPICure study. Arch Dis Child Fetal Neonatal Ed. (2021) 106:149–55. 10.1136/archdischild-2020-319328 32796060 PMC7116791

[20] Ali GB Bui DS Lodge CJ Waidyatillake NT Perret JL Sun C. Infant body mass index trajectories, and asthma & lung function. J Allergy Clin Immunol. (2021). 10.1016/j.jaci.2021.02.020 33662371

[21] WHO. World Health Organization Child Growth Standards. (2018). Available online at: https://www.who.int/toolkits/child-growth-standards/standards/body-mass-index-for-age-bmi-for-age (accessed April 27, 2006).

[22] Hou X Lu J Weng J Ji L Shan Z Liu J. Impact of waist circumference and body mass index on risk of cardiometabolic disorder and cardiovascular disease in Chinese adults: a national diabetes and metabolic disorders survey. PLoS ONE. (2013) 8:e57319. 10.1371/journal.pone.0057319 23520466 PMC3592870

[23] Lee AC Katz J Blencowe H Cousens S Kozuki N Vogel JP. National and regional estimates of term and preterm babies born small for gestational age in 138 low-income and middle-income countries in 2010. Lancet Glob Health. (2013) 1:e26–36. 10.1016/S2214-109X(13)70006-8 25103583 PMC4221634

[24] American Academy Of Pediatrics Committee On F, Newborn, American College Of, O Gynecologists Committee On Obstetric P. The Apgar score. Pediatrics. (2015) 136:819–22. 10.1542/peds.2015-2651 26416932

[25] Ong KK Preece MA Emmett PM Ahmed ML Dunger DB Team AS. Size at birth and early childhood growth in relation to maternal smoking, parity and infant breast-feeding: longitudinal birth cohort study and analysis. Pediatr Res. (2002) 52:863–7. 10.1203/00006450-200212000-00009 12438662

[26] Giles LC Whitrow MJ Davies MJ Davies CE Rumbold AR Moore VM. Growth trajectories in early childhood, their relationship with antenatal and postnatal factors, and development of obesity by age 9 years: results from an Australian birth cohort study. Int J Obes. (2015) 39:1049–56. 10.1038/ijo.2015.42 26008137

[27] Catalano PM Shankar K. Obesity and pregnancy: mechanisms of short term and long term adverse consequences for mother and child. BMJ. (2017) 356:j1. 10.1136/bmj.j1 28179267 PMC6888512

[28] Choi HJ Lee HJ Jang HB Park JY Kang JH Park KH. Effects of maternal education on diet, anemia, and iron deficiency in Korean school-aged children. BMC Public Health. (2011) 11:870. 10.1186/1471-2458-11-870 22087564 PMC3250969

[29] Keag OE Norman JE Stock SJ. Long-term risks and benefits associated with cesarean delivery for mother, baby, and subsequent pregnancies: systematic review and meta-analysis. PLoS Med. (2018) 15:e1002494. 10.1371/journal.pmed.1002494 29360829 PMC5779640

[30] Yuan C Gaskins AJ Blaine AI Zhang C Gillman MW Missmer SA. Association between cesarean birth and risk of obesity in offspring in childhood, adolescence, and early adulthood. JAMA Pediatr. (2016) 170:e162385. 10.1001/jamapediatrics.2016.2385 27599167 PMC5854473

[31] Ajslev TA Andersen CS Gamborg M Sorensen TI Jess T. Childhood overweight after establishment of the gut microbiota: the role of delivery mode, pre-pregnancy weight and early administration of antibiotics. Int J Obes. (2011) 35:522–9. 10.1038/ijo.2011.27 21386800

[32] Gronlund MM Lehtonen OP Eerola E Kero P. Fecal microflora in healthy infants born by different methods of delivery: permanent changes in intestinal flora after cesarean delivery. J Pediatr Gastroenterol Nutr. (1999) 28:19–25. 10.1097/00005176-199901000-00007 9890463

[33] Hyde MJ Mostyn A Modi N Kemp PR. The health implications of birth by Caesarean section. Biol Rev Camb Philos Soc. (2012) 87:229–43. 10.1111/j.1469-185X.2011.00195.x 21815988

[34] Catalano PM Farrell K Thomas A Huston-Presley L Mencin P de Mouzon SH. Perinatal risk factors for childhood obesity and metabolic dysregulation. Am J Clin Nutr. (2009) 90:1303–13. 10.3945/ajcn.2008.27416 19759171 PMC2762159

[35] Barker DJ. The origins of the developmental origins theory. J Intern Med. (2007) 261:412–7. 10.1111/j.1365-2796.2007.01809.x 17444880

[36] Kim SY Sharma AJ Callaghan WM. Gestational diabetes and childhood obesity: what is the link? Curr Opin Obstet Gynecol. (2012) 24:376–81. 10.1097/GCO.0b013e328359f0f4 23000698 PMC4392761

[37] Ortega-Garcia JA Kloosterman N Alvarez L Tobarra-Sanchez E Carceles-Alvarez A Pastor-Valero R. Full breastfeeding and obesity in children: a prospective study from birth to 6 years. Child Obes. (2018) 14:327–37. 10.1089/chi.2017.0335 29912590 PMC6066191

[38] Ma J Qiao Y Zhao P Li W Katzmarzyk PT Chaput JP. Breastfeeding and childhood obesity: a 12-country study. Matern Child Nutr. (2020) 16:e12984. 10.1111/mcn.12984 32141229 PMC7296809

[39] Rzehak P Oddy WH Mearin ML Grote V Mori TA Szajewska H. Infant feeding and growth trajectory patterns in childhood and body composition in young adulthood. Am J Clin Nutr. (2017) 106:568–80. 10.3945/ajcn.116.140962 28659295

[40] Forbes JD Azad MB Vehling L Tun HM Konya TB Guttman DS. Association of exposure to formula in the hospital and subsequent infant feeding practices with gut microbiota and risk of overweight in the first year of life. JAMA Pediatr. (2018) 172:e181161. 10.1001/jamapediatrics.2018.1161 29868719 PMC6137517

