# Supplementary material for: BMI Trajectories During the First 2 Years, and Their Associations With Infant Overweight/Obesity: A Registered Based Cohort Study in Taizhou, China
Source: Front Pediatr. 2021 May 12;9:665655. doi: 10.3389/fped.2021.665655 (PMC8149948; doi:10.3389/fped.2021.665655)
Supplement: Supplementary file 1 [file Data_Sheet_1.docx]

Supplementary Materials

# Supplementary Tables

Supplementary Table 1.1: Parameter estimates of the models with 1 to 5 trajectories.

| Nb. Latent classes | Polynomial degree | Log-Likelihood | BIC | Participants per class (%) | Mean posterior probabilities (%) | Posterior probabilities > 0.7 (%) |
| --- | --- | --- | --- | --- | --- | --- |
| 1 | Linear | -211429.90 | -211444.60 | 100.00 | 100.00 | 100.00 |
| 1 | Quadratic | -191501.60 | -191521.30 | 100.00 | 100.00 | 100.00 |
| 1 | Cubic | -183031.00 | -183055.60 | 100.00 | 100.00 | 100.00 |
| 2 | Linear | -207589.00 | -207623.50 | 67.03/32.97 | 88.22/80.79 | 84.67/71.32 |
| 2 | Quadratic | -184019.50 | -184063.90 | 63.67/36.33 | 91.48/87.62 | 89.51/83.26 |
| 2 | Cubic | -172289.80 | -172344.00 | 61.78/38.22 | 93.03/90.22 | 91.74/87.08 |
| 3 | Linear | -206900.10 | -206954.30 | 44.73/10.83/44.44 | 79.82/71.60/69.89 | 69.63/50.34/46.42 |
| 3 | Quadratic | -182245.60 | -182314.50 | 37.61/53.91/8.48 | 85.19/83.56/83.87 | 79.80/80.54/76.04 |
| 3 | Cubic | -169215.90 | -169299.70 | 36.21/53.15/10.64 | 87.91/86.75/86.98 | 83.85/84.40/80.96 |
| 4 | Linear | -206500.20 | -206574.10 | 32.35/16.68/49.39/1.58 | 76.29/68.96/69.98/73.95 | 62.56/45.89/48.20/56.48 |
| 4 | Quadratic | -182245.60 | -182339.20 | 37.61/53.91/8.48/0.00 | 85.19/83.56/83.87/0.00 | 79.80/80.54/76.04/0.00 |
| 4 | Cubic | -167710.30 | -167823.70 | 35.09/32.69/11.58/20.64 | 87.06/78.01/86.40/75.63 | 81.82/67.45/79.96/61.15 |
| 5 | Linear | -206132.80 | -206226.50 | 29.59/34.51/7.92/26.58/1.40 | 57.97/75.95/66.27/64.56/71.92 | 18.23/61.90/42.08/36.80/55.06 |
| 5 | Quadratic | -180185.40 | -180303.70 | 26.83/22.86/13.01/2.12/35.19 | 80.46/70.22/74.79/83.80/71.31 | 70.95/50.11/59.00/75.68/51.96 |
| 5 | Cubic | -166421.60 | -166564.50 | 23.48/31.34/22.66/19.92/2.59 | 84.45/75.94/82.53/75.04/89.79 | 77.16/63.19/75.64/59.80/86.84 |

Reported are: the number of latent class considered, the polynomial form of the model, the maximum Log-Likelihood , the Bayesian information Criterion (BIC), and for models with 2 or more classes, the a-posteriori classification of subjects in each class (%), the mean of posterior probabilities (%) in each latent class, and the % of subjects classified in each class with a posterior probability above 0.7.

Supplementary Table 1.2: Intercept and slope of each trajectory group for the 3 trajectories with cubic equation.

|  | **Lower trajectory (*p*)** | **Middle trajectory (*p*)** | **Upper trajectory (*p*)** |
| --- | --- | --- | --- |
| Intercept | 12.96691 (<0.001) | 13.75068 (<0.001) | 14.44042 (<0.001) |
| Slope |  |  |  |
| Linear | 1.26074 (<0.001) | 1.62508 (<0.001) | 2.08869 (<0.001) |
| Quadratic | -0.12578 (<0.001) | -0.17048 (<0.001) | -0.21628 (<0.001) |
| Cubic | 0.00350 (<0.001) | 0.00496 (<0.001) | 0.00618 (<0.001) |

Supplementary Table 2.1: Factors associated with BMI development trajectory groups in boys.

| **Variables, n (%)or mean (SD)** | **Total population** | **Lower trajectory** | **Middle trajectory** | **Upper trajectory** | ***P*-value** |
| --- | --- | --- | --- | --- | --- |
|  | **N=9973** | **n=3697** | **n=5400** | **n=876** |  |
| **Maternal characteristics** |  |  |  |  |  |
| ***Maternal demographics*** |  |  |  |  |  |
| Age (years) (mean±SD) | 30.64±5.23 | 30.57±5.33 | 30.68±5.20 | 30.72±4.98 | 0.549 |
| Education, n (%) |  |  |  |  | 0.565 |
| Primary school or below | 613 (6.15%) | 241 (6.52%) | 326 (6.04%) | 46 (5.25%) |  |
| Junior school | 2676 (26.83%) | 992 (26.83%) | 1437 (26.61%) | 247 (28.20%) |  |
| High school | 2617 (26.24%) | 966 (26.13%) | 1408 (26.07%) | 243 (27.74%) |  |
| University or above | 4067 (40.78%) | 1498 (40.52%) | 2229 (41.28%) | 340 (38.81%) |  |
| Parity, n (%) |  |  |  |  | **0.004** |
| Primiparous | 3480 (34.89%) | 1352 (36.57%) | 1857 (34.39%) | 271 (30.94%) |  |
| Multiparous | 6493 (65.11%) | 2345 (63.43%) | 3543 (65.61%) | 605 (69.06%) |  |
| Abortion times, n (%) |  |  |  |  |  |
| Induced |  |  |  |  | 0.294 |
| 0 | 6890 (69.09%) | 2560 (69.25%) | 3730 (69.07%) | 600 (68.49%) |  |
| 1 | 1754 (17.59%) | 618 (16.72%) | 976 (18.07%) | 160 (18.26%) |  |
| 2+ | 1329 (13.32%) | 519 (14.04%) | 694 (12.85%) | 116 (13.24%) |  |
| Spontaneous |  |  |  |  | 0.672 |
| 0 | 9341 (93.66%) | 3460 (93.59%) | 5061 (93.72%) | 820 (93.61%) |  |
| 1 | 531 (5.33%) | 204 (5.52%) | 278 (5.15%) | 49 (5.59%) |  |
| 2+ | 101 (1.01%) | 33 (0.89%) | 61 (1.13%) | 7 (0.80%) |  |
| ***Prenatal characteristics*** |  |  |  |  |  |
| BMI at early stage of pregnancy, n (%) | |  |  |  | **<0.001** |
| BMI < 18.5 kg/m² | 1778 (17.83%) | 872 (23.59%) | 819 (15.17%) | 87 (9.93%) |  |
| BMI :18.5-23.9 kg/m² | 5741 (57.57%) | 2100 (56.80%) | 3161 (58.54%) | 480 (54.80%) |  |
| BMI :24.0-27.9 kg/m² | 1934 (19.39%) | 590 (15.96%) | 1113 (20.61%) | 231 (26.37%) |  |
| BMI ≥28.0 kg/m² | 520 (5.21%) | 135 (3.65%) | 307 (5.68%) | 78 (8.90%) |  |
| Smoking during pregnancy, n (%) | 11 (0.11%) | 2 (0.05%) | 8 (0.15%) | 1 (0.11%) | 0.414 |
| Drinking during pregnancy, n (%) | 23 (0.23%) | 5 (0.14%) | 16 (0.30%) | 2 (0.23%) | 0.290 |
| Gestational week (mean±SD) | 38.93±1.25 | 38.86±1.33 | 38.96±1.21 | 39.04±1.14 | **<0.001** |
| Gestational complications, n (%) |  |  |  |  |  |
| GDM | 1512 (15.16%) | 517 (13.98%) | 827 (15.31%) | 168 (19.18%) | **0.001** |
| Gestational hypertension | 151 (1.51%) | 58 (1.57%) | 80 (1.48%) | 13 (1.48%) | 0.943 |
| Anemia | 1573 (15.77%) | 597 (16.15%) | 851 (15.76%) | 125 (14.27%) | 0.390 |
| Thyroid disease | 1401 (14.05%) | 480 (12.98%) | 766 (14.19%) | 155 (17.69%) | **0.001** |
| **Infant characteristics** |  |  |  |  |  |
| ***Neonatal characteristics*** |  |  |  |  |  |
| SGA, n (%) | 621 (6.23%) | 436 (11.79%) | 173 (3.20%) | 12 (1.37%) | **<0.001** |
| Mode of delivery, n (%) |  |  |  |  | **<0.001** |
| Vaginal delivery | 4759 (47.72%) | 1956 (52.91%) | 2460 (45.56%) | 343 (39.16%) |  |
| Caesarean | 5214 (52.28%) | 1741 (47.09%) | 2940 (54.44%) | 533 (60.84%) |  |
| Apgar 1 min ≥8, n (%) | 9867 (98.94%) | 3644 (98.57%) | 5353 (99.13%) | 870 (99.32%) | **0.019** |
| Apgar 5 mins ≥8, n (%) | 9945 (99.72%) | 3683 (99.62%) | 5389 (99.80%) | 873 (99.66%) | 0.282 |
| Birth weight (g) (mean±SD) | 3376.56±398.99 | 3196.47±384.37 | 3452.91±404.42 | 3665.98±425.02 | **<0.001** |
| ***Postnatal characteristics*** |  |  |  |  |  |
| Exclusive breastfeeding, n (%) | 6229 (62.46%) | 2261 (61.16%) | 3405 (63.06%) | 563 (64.27%) | 0.095 |
| Daily intake of vitamin D (IU/d), n (%) |  |  |  |  | 0.665 |
| 0≤vitamin D≤400 | 8573 (85.96%) | 3170 (85.75%) | 4648 (86.07%) | 755 (86.19%) |  |
| 400<vitamin D≤600 | 1161 (11.64%) | 428 (11.58%) | 629 (11.65%) | 104 (11.87%) |  |
| 600<vitamin D | 239 (2.40%) | 99 (2.68%) | 123 (2.28%) | 17 (1.94%) |  |
| Outdoor activity duration (hour), n (%) | |  |  |  | 0.144 |
| 0-1 | 226 (2.26%) | 83 (2.25%) | 123 (2.28%) | 20 (2.28%) |  |
| 1-2 | 2835 (28.43%) | 1019 (27.56%) | 1536 (28.44%) | 280 (31.97%) |  |
| ≥2 | 6912 (69.31%) | 2595 (70.19%) | 3741 (69.28%) | 576 (65.75%) |  |
| Overweight, n(%) | 1691 (16.96%) | 66 (1.79%) | 1408 (26.07%) | 217 (24.77%) | **<0.001** |
| Obesity, n(%) | 1094 (10.97%) | 4 (0.11%) | 493 (9.13%) | 597 (68.15%) | **<0.001** |

*Note：*Boldface indicates statistical significance (*p*<0.05).

Supplementary Table 2.2: Factors associated with BMI development trajectory groups in girls.

| **Variables, n (%)or mean (SD)** | **Total population** | **Lower trajectory** | **Middle trajectory** | **Upper trajectory** | ***P*-value** |
| --- | --- | --- | --- | --- | --- |
|  | **N=9081** | **n=3168** | **n=4892** | **n=1021** |  |
| **Maternal characteristics** |  |  |  |  |  |
| ***Maternal demographics*** |  |  |  |  |  |
| Age (years) (mean±SD) | 30.58±5.21 | 30.50±5.22 | 30.64±5.25 | 30.52±4.96 | 0.504 |
| Education, n (%) |  |  |  |  | 0.525 |
| Primary school or below | 588 (6.48%) | 225 (7.10%) | 298 (6.09%) | 65 (6.36%) |  |
| Junior school | 2383 (26.24%) | 813 (25.66%) | 1306 (26.70%) | 264 (25.86%) |  |
| High school | 2275 (25.05%) | 784 (24.75%) | 1243 (25.41%) | 248 (24.29%) |  |
| University or above | 3835 (42.23%) | 1346 (42.49%) | 2045 (41.80%) | 444 (43.49%) |  |
| Parity, n (%) |  |  |  |  | **0.001** |
| Primiparous | 3256 (35.86%) | 1214 (38.32%) | 1698 (34.71%) | 344 (33.69%) |  |
| Multiparous | 5825 (64.14%) | 1954 (61.68%) | 3194 (65.29%) | 677 (66.31%) |  |
| Abortion times, n (%) |  |  |  |  |  |
| Induced |  |  |  |  | 0.195 |
| 0 | 6250 (68.83%) | 2225 (70.23%) | 3315 (67.76%) | 710 (69.54%) |  |
| 1 | 1649 (18.15%) | 554 (17.49%) | 918 (18.77%) | 177 (17.34%) |  |
| 2+ | 1182 (13.02%) | 389 (12.28%) | 659 (13.47%) | 134 (13.12%) |  |
| Spontaneous |  |  |  |  | 0.469 |
| 0 | 8513 (93.74%) | 2986 (94.25%) | 4566 (93.34%) | 961(94.12%) |  |
| 1 | 462 (5.09%) | 151 (4.77%) | 262 (5.35%) | 49 (4.80%) |  |
| 2+ | 106 (1.17%) | 31 (0.98%) | 64 (1.31%) | 11 (1.08%) |  |
| ***Prenatal characteristics*** |  |  |  |  |  |
| BMI at early stage of pregnancy, n (%) |  |  |  |  | **<0.001** |
| BMI < 18.5 kg/m² | 1634 (17.99%) | 786 (24.81%) | 733 (14.98%) | 115 (11.26%) |  |
| BMI :18.5-23.9 kg/m² | 5301 (58.38%) | 1854 (58.52%) | 2887 (59.02%) | 560 (54.85%) |  |
| BMI :24.0-27.9 kg/m² | 1697 (18.69%) | 427 (13.48%) | 1001 (20.46%) | 269 (26.35%) |  |
| BMI ≥28.0 kg/m² | 449 (4.94%) | 101 (3.19%) | 271 (5.54%) | 77 (7.54%) |  |
| Smoking during pregnancy, n (%) | 9 (0.10%) | 6 (0.19%) | 2 (0.04%) | 1 (0.10%) | 0.118 |
| Drinking during pregnancy, n (%) | 11 (0.12%) | 4 (0.13%) | 4 (0.08%) | 3 (0.29%) | 0.207 |
| Gestational week (mean±SD) | 39.05±1.23 | 38.96±1.30 | 39.10±1.18 | 39.12±1.18 | **<0.001** |
| Gestational complications, n (%) |  |  |  |  |  |
| GDM | 1283 (14.13%) | 451 (14.24%) | 708 (14.47%) | 124 (12.14%) | 0.148 |
| Gestational hypertension | 149 (1.64%) | 61 (1.93%) | 75 (1.53%) | 13 (1.27%) | 0.247 |
| Anemia | 1478 (16.28%) | 507 (16.00%) | 796 (16.27%) | 175 (17.14%) | 0.694 |
| Thyroid disease | 1217 (13.40%) | 428 (13.51%) | 671 (13.72%) | 118 (11.56%) | 0.179 |
| **Infant characteristics** |  |  |  |  |  |
| ***Neonatal characteristics*** |  |  |  |  |  |
| SGA, n (%) | 1037 (11.42%) | 677 (21.37%) | 330 (6.75%) | 30 (2.94%) | **<0.001** |
| Mode of delivery, n (%) |  |  |  |  | **<0.001** |
| Vaginal delivery | 4573 (50.36%) | 1682 (53.09%) | 2439 (49.86%) | 452 (44.27%) |  |
| Caesarean | 4508 (49.64%) | 1486 (46.91%) | 2453 (50.14%) | 569 (55.73%) |  |
| Apgar 1 min ≥8, n (%) | 9013 (99.25%) | 3139 (99.08%) | 4859 (99.33%) | 1015 (99.41%) | 0.386 |
| Apgar 5 mins ≥8, n (%) | 9065 (99.82%) | 3161 (99.78%) | 4885 (99.86%) | 1019 (99.80%) | 0.709 |
| Birth weight (g) (mean±SD) | 3258.51±386.88 | 3079.82±367.00 | 3328.08±393.04 | 3479.62±416.15 | **<0.001** |
| Postnatal characteristics |  |  |  |  |  |
| Exclusive breastfeeding, n (%) | 5985 (65.91%) | 2041 (64.43%) | 3231 (66.05%) | 713 (69.83%) | **0.006** |
| Daily intake of vitamin D (IU/d), n (%) |  |  |  |  | 0.295 |
| 0≤vitamin D≤400 | 7850 (86.45%) | 2724 (85.98%) | 4248 (86.84%) | 878 (86.00%) |  |
| 400<vitamin D≤600 | 1032 (11.36%) | 361 (11.40%) | 551 (11.26%) | 120 (11.75%) |  |
| 600<vitamin D | 199 (2.19%) | 83 (2.62%) | 93 (1.90%) | 23 (2.25%) |  |
| Outdoor activity duration (hour), n (%) |  |  |  |  | **0.015** |
| 0-1 | 198 (2.18%) | 58 (1.83%) | 114 (2.33%) | 26 (2.55%) |  |
| 1-2 | 2556 (28.15%) | 843 (26.61%) | 1396 (28.54%) | 317 (31.05%) |  |
| ≥2 | 6327(69.67%) | 2267 (71.56%) | 3382 (69.13%) | 678 (66.40%) |  |
| Overweight, n (%) | 1303 (14.35%) | 32(1.01%) | 901 (18.42%) | 370 (36.24%) | **<0.001** |
| Obesity, n (%) | 727 (8.01%) | 2 (0.06%) | 208 (4.25%) | 517 (50.64%) | **<0.001** |

## *Note：*Boldface indicates statistical significance (*p*<0.05).

Supplementary Table 3.1: Association between prenatal/early life factors with middle and upper BMI trajectories in boys (a comparison to lower BMI trajectories).

| **Variables** | **Middle trajectory** | **Upper trajectory** |
| --- | --- | --- |
|  | **OR (95%CI)** | **OR (95%CI)** |
| **Maternal characteristics** |  |  |
| ***Maternal demographics*** |  |  |
| Age (years) |  |  |
| <18 | 0.66 (0.25-1.76) | 0.49 (0.06-3.96) |
| 18-24 | **0.87 (0.77-0.99)** | 0.88 (0.71-1.11) |
| 25-34 | 1.00 (reference) | 1.00 (reference) |
| ≥35 | 0.92 (0.83-1.01) | **0.81 (0.68-0.97)** |
| Education |  |  |
| Primary school or below | 1.00 (reference) | 1.00 (reference) |
| Junior school | 1.07 (0.89-1.29) | 1.30 (0.92-1.84) |
| High school | 1.08 (0.90-1.30) | 1.32 (0.93-1.86) |
| University or above | 1.10 (0.92-1.32) | 1.19 (0.85-1.67) |
| ***Prenatal characteristics*** |  |  |
| BMI at early stage of pregnancy |  |  |
| BMI < 18.5 kg/m² | **0.62 (0.56-0.70)** | **0.44 (0.34-0.56)** |
| BMI :18.5-23.9 kg/m² | 1.00 (reference) | 1.00 (reference) |
| BMI :24.0-27.9 kg/m² | **1.25 (1.12-1.41)** | **1.71 (1.43-2.05)** |
| BMI ≥28.0 kg/m² | **1.51 (1.23-1.86)** | **2.53 (1.88-3.40)** |
| Parity |  |  |
| Primiparous | 1.00 (reference) | 1.00 (reference) |
| Multiparous | **1.10 (1.01-1.20)** | **1.29 (1.10-1.51)** |
| Smoking during pregnancy |  |  |
| No | 1.00 (reference) | 1.00 (reference) |
| Yes | 2.74 (0.58-12.92) | 2.11 (0.19-23.31) |
| Drinking during pregnancy |  |  |
| No | 1.00 (reference) | 1.00 (reference) |
| Yes | 2.19 (0.80-5.98) | 1.69 (0.33-8.71) |
| ***Gestational complications*** |  |  |
| GDM |  |  |
| No | 1.00 (reference) | 1.00 (reference) |
| Yes | 1.11 (0.99-1.25) | **1.46 (1.21-1.77)** |
| Gestational hypertension |  |  |
| No | 1.00 (reference) | 1.00 (reference) |
| Yes | 0.94 (0.67-1.33) | 0.95 (0.52-1.73) |
| Anemia |  |  |
| No | 1.00 (reference) | 1.00 (reference) |
| Yes | 0.97 (0.87-1.09) | 0.86 (0.70-1.07) |
| Thyroid disease |  |  |
| No | 1.00 (reference) | 1.00 (reference) |
| Yes | 1.11 (0.98-1.25) | **1.44 (1.18-1.76)** |
| **Infant characteristics** |  |  |
| ***Neonatal characteristics*** |  |  |
| SGA |  |  |
| No | 1.00 (reference) | 1.00 (reference) |
| Yes | **0.25 (0.21-0.30)** | **0.10 (0.06-0.19)** |
| Mode of delivery |  |  |
| Cesarean | 1.00 (reference) | 1.00 (reference) |
| Vaginal delivery | **0.75 (0.69-0.81)** | **0.57 (0.49-0.67)** |
| Apgar 1 min |  |  |
| ≥8 | 1.00 (reference) | 1.00 (reference) |
| <8 | **0.60 (0.41-0.90)** | 0.48 (0.20-1.11) |
| Apgar 5 mins |  |  |
| ≥8 | 1.00 (reference) | 1.00 (reference) |
| <8 | 0.54 (0.24-1.18) | 0.90 (0.26-3.15) |
| ***Postnatal characteristics*** |  |  |
| Exclusive breastfeeding |  |  |
| No | 1.00 (reference) | 1.00 (reference) |
| Yes | 1.08 (0.99-1.18) | 1.14 (0.98-1.33) |
| Vitamin D (IU/d) |  |  |
| 0 ≤ Vitamin D ≤ 400 | 1.00 (reference) | 1.00 (reference) |
| 400 < Vitamin D ≤600 | 1.00 (0.88-1.14) | 1.02 (0.81-1.28) |
| Vitamin D > 600 | 0.85 (0.65-1.11) | 0.72 (0.43-1.21) |
| Outdoor activity duration (hours/d) |  |  |
| 0-1 | 1.00 (reference) | 1.00 (reference) |
| 1-2 | 1.02 (0.76-1.36) | 1.14 (0.69-1.89) |
| ≥2 | 0.97 (0.73-1.29) | 0.92 (0.56-1.51) |

*Note：*Boldface indicates statistical significance (*p*<0.05).

Supplementary Table 3.2: Association between prenatal/early life factors with middle and upper BMI trajectories in girls (a comparison to lower BMI trajectories).

| **Variables** | **Middle trajectory** | **Upper trajectory** |
| --- | --- | --- |
|  | **OR (95%CI)** | **OR (95%CI)** |
| **Maternal characteristics** |  |  |
| ***Maternal demographics*** |  |  |
| Age (year) |  |  |
| <18 | 1.31(0.45-3.84) | <0.01 (<0.01->999.99) |
| 18-24 | 1.02 (0.89-1.17) | 0.88 (0.71-1.10) |
| 25-34 | 1.00 (reference) | 1.00 (reference) |
| ≥35 | 1.04 (0.94-1.16) | 0.87 (0.73-1.04) |
| Education |  |  |
| Primary school or below | 1.00 (reference) | 1.00 (reference) |
| Junior school | 1.21 (0.99-1.47) | 1.12 (0.83-1.53) |
| High school | 1.20 (0.99-1.45) | 1.10 (0.80-1.49) |
| University or above | 1.15 (0.95-1.38) | 1.14 (0.85-1.54) |
| ***Prenatal characteristics*** |  |  |
| BMI at early stage of pregnancy |  |  |
| BMI < 18.5 kg/m² | **0.60 (0.53-0.67)** | **0.48 (0.39-0.60)** |
| BMI :18.5-23.9 kg/m² | 1.00 (reference) | 1.00 (reference) |
| BMI :24.0-27.9 kg/m² | **1.51 (1.33-1.71)** | **2.09 (1.74-2.50)** |
| BMI ≥28.0 kg/m² | **1.72 (1.36-2.18)** | **2.52 (1.85-3.45)** |
| Parity |  |  |
| Primiparous | 1.00 (reference) | 1.00 (reference) |
| Multiparous | **1.17 (1.07-1.28)** | **1.22 (1.05-1.42)** |
| Smoking during pregnancy |  |  |
| No | 1.00 (reference) | 1.00 (reference) |
| Yes | 0.22 (0.04-1.07) | 0.52 (0.06-4.30) |
| Drinking during pregnancy |  |  |
| No | 1.00 (reference) | 1.00 (reference) |
| Yes | 0.65 (0.16-2.59) | 2.33 (0.52-10.44) |
| ***Gestational complications*** |  |  |
| GDM |  |  |
| No | 1.00 (reference) | 1.00 (reference) |
| Yes | 1.02 (0.90-1.16) | 0.83 (0.67-1.03) |
| Gestational hypertension |  |  |
| No | 1.00 (reference) | 1.00 (reference) |
| Yes | 0.79 (0.56-1.12) | 0.66 (0.36-1.20) |
| Anemia |  |  |
| No | 1.00 (reference) | 1.00 (reference) |
| Yes | 1.02 (0.90-1.15) | 1.09 (0.90-1.31) |
| Thyroid disease |  |  |
| No | 1.00 (reference) | 1.00 (reference) |
| Yes | 1.02 (0.89-1.16) | 0.84 (0.67-1.04) |
| **Infant characteristics** |  |  |
| ***Neonatal characteristics*** |  |  |
| SGA |  |  |
| No | 1.00 (reference) | 1.00 (reference) |
| Yes | **0.27 (0.23-0.31)** | **0.11 (0.08-0.16)** |
| Mode of delivery |  |  |
| Cesarean | 1.00 (reference) | 1.00 (reference) |
| Vaginal delivery | **0.88 (0.80-0.96)** | **0.70 (0.61-0.81)** |
| Apgar 1 min |  |  |
| ≥8 | 1.00 (reference) | 1.00 (reference) |
| <8 | 0.74 (0.45-1.21) | 0.64 (0.27-1.55) |
| Apgar 5 mins |  |  |
| ≥8 | 1.00 (reference) | 1.00 (reference) |
| <8 | 0.65 (0.23-1.85) | 0.89 (0.18-4.27) |
| ***Postnatal characteristics*** |  |  |
| Exclusive breastfeeding |  |  |
| No | 1.00 (reference) | 1.00 (reference) |
| Yes | 1.07 (0.98-1.18) | **1.28 (1.10-1.49)** |
| Vitamin D (IU/d) |  |  |
| 0 ≤ Vitamin D ≤ 400 | 1.00 (reference) | 1.00 (reference) |
| 400 < Vitamin D ≤600 | 0.98 (0.85-1.13) | 1.03 (0.83-1.29) |
| Vitamin D > 600 | **0.72 (0.53-0.97)** | 0.86 (0.54-1.37) |
| Outdoor activity duration (hours/d) |  |  |
| 0-1 | 1.00 (reference) | 1.00 (reference) |
| 1-2 | 0.84 (0.61-1.17) | 0.84 (0.52-1.36) |
| ≥2 | 0.76 (0.55-1.05) | 0.67 (0.42-1.07) |

*Note：*Boldface indicates statistical significance (*p*<0.05).

Supplementary Table 4.1: Association between childhood obesity/overweight and BMI trajectories in boys by log-binomial regression.

|  | **Lower trajectory** | **Middle trajectory** | **Upper trajectory** |
| --- | --- | --- | --- |
|  | **n=3697** | **n=5400** | **n=876** |
|  | **PR(95%CI)** | **PR(95%CI)** | **PR(95%CI)** |
| **Overweight/Obesity (n, %)** | 70 (1.89%) | 1901 (35.20%) | 814 (92.92%) |
| **Model 1*** | 1.00 (reference) | 18.59 (14.83-23.74) | 49.08 (39.23-62.54) |
| **Model 2*** | 1.00 (reference) | 18.59 (14.83-23.73) | 49.08 (39.24-62.54) |
| **Model 3*** | 1.00 (reference) | 4.07 (3.99-6.11) | 7.93 (7.35-12.51) |
| **Model 4*** | 1.00 (reference) | 2.72 (2.72-14.46) | 3.68 (3.61-3.68) |
| *Model 1 was unadjusted; *Model 2 was adjusted for age, gender, education; *Model 3 was additionally controlled for maternal factors, including parity, early pregnancy BMI, GDM, gestational hypertension, based on model 2; *Model 4 was additionally controlled for characteristics of infants, including mode of delivery, outdoor activity duration, maternal feeding styles, SGA, infants' age of their last measurement and intake of vitamin D based on model 3. | | | |

Table S4.2: Association between childhood obesity/overweight and BMI trajectories in girls by log-binomial regression.

|  | **Lower trajectory** | **Middle trajectory** | **Upper trajectory** |
| --- | --- | --- | --- |
|  | **n=3168** | **n=4892** | **n=1021** |
|  | **PR(95%CI)** | **PR(95%CI)** | **PR(95%CI)** |
| **Overweight/Obesity (n, %)** | 34 (1.07%) | 1109 (22.67%) | 887 (86.88%) |
| **Model 1*** | 1.00 (reference) | 21.12 (15.33-30.22) | 80.95 (58.94-115.47) |
| **Model 2*** | 1.00 (reference) | 21.13 (15.33-30.22) | 80.76 (58.81-115.21) |
| **Model 3*** | 1.00 (reference) | 21.00 (15.24-30.04) | 79.74 (58.04-113.77) |
| **Model 4*** | 1.00 (reference) | 3.31 (3.31-3.31) | 4.56 (4.55-4.56) |
| *Model 1 was unadjusted; *Model 2 was adjusted for age, gender, education; *Model 3 was additionally controlled for maternal factors, including parity, early pregnancy BMI, GDM, gestational hypertension, based on model 2; *Model 4 was additionally controlled for characteristics of infants, including mode of delivery, outdoor activity duration, maternal feeding styles, SGA, infants' age of their last measurement and intake of vitamin D based on model 3. | | | |

**Supplementary Figures**


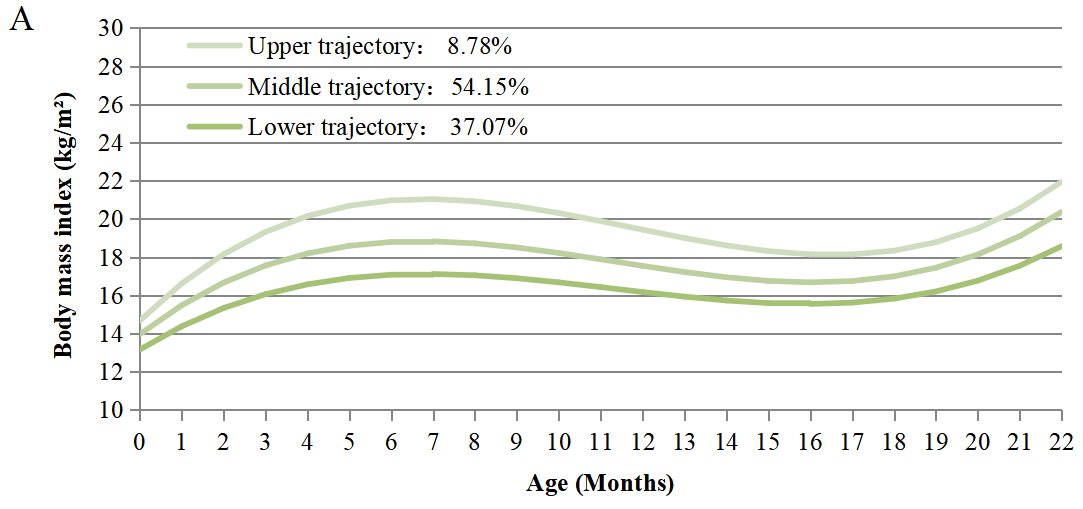


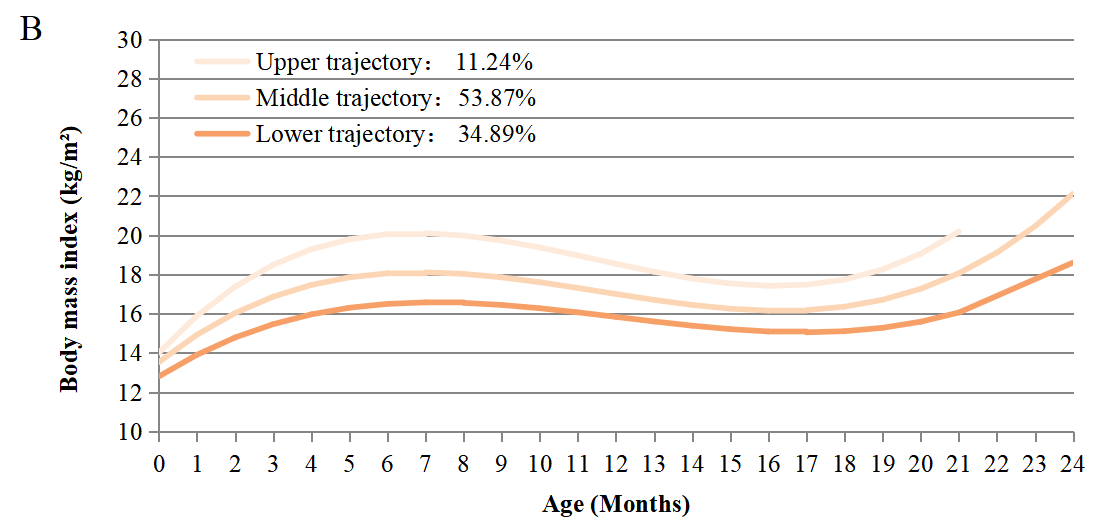


Figure S1: (A) BMI trajectories from birth to 2 years old identified from the LCGMM in boys. (B) BMI trajectories from birth to 2 years old identified from the LCGMM in girls.
